# Exploring the impact of pre-anastomosis cerebral microcirculation on cerebral hyperperfusion syndrome in superficial temporal artery-middle cerebral artery bypass surgery of moyamoya disease

**DOI:** 10.1117/1.NPh.11.3.035008

**Published:** 2024-09-04

**Authors:** Wenting Zhu, Tianshu Tao, Jiachi Hong, Ruolan Li, Minghui Ma, Jianjian Zhang, Jincao Chen, Jinling Lu, Pengcheng Li

**Affiliations:** a Huazhong University of Science and Technology, Britton Chance Center and MoE Key Laboratory for Biomedical Photonics, Wuhan National Laboratory for Optoelectronics, Wuhan, China; b Zhongnan Hospital of Wuhan University, Wuhan University, Department of Neurosurgery, Wuhan, China; c Hainan University, School of Biomedical Engineering, State Key Laboratory of Digital Medical Engineering, Sanya, China; d Huazhong University of Science and Technology, Advanced Biomedical Imaging Facility, Wuhan, China

**Keywords:** laser speckle contrast imaging, moyamoya disease, STA-MCA bypass, cerebral hyperperfusion syndrome, baseline cerebral blood flow

## Abstract

**Significance:**

Cerebral hyperperfusion syndrome (CHS), characterized by neurologic deficits due to postoperative high cerebral perfusion, is a serious complication of superficial temporal artery-middle cerebral artery (STA-MCA) surgery for moyamoya disease (MMD).

**Aim:**

We aim to clarify the importance of assessing pre-anastomosis cerebral microcirculation levels by linking the onset of CHS to pre- and post-anastomosis hemodynamics.

**Approach:**

Intraoperative laser speckle contrast imaging (LSCI) measured changes in regional cerebral blood flow (rCBF) and regional blood flow structuring (rBFS) within the cerebral cortical microcirculation of 48 adults with MMD.

**Results:**

Following anastomosis, all MMD patients exhibited a significant increase in rCBF (279.60%±120.00%, p<0.001). Changes in rCBF and rBFS showed a negative correlation with their respective baseline levels (rCBF, p<0.001; rBFS, p=0.005). Baseline rCBF differed significantly between CHS and non-CHS groups (p=0.0049). The areas under the receiver operating characteristic (ROC) curve for baseline rCBF was 0.753. Hemorrhagic MMD patients showed higher baseline rCBF than ischemic patients (p=0.036), with a marked correlation between pre- and post-anastomosis rCBF in hemorrhagic cases (p=0.003), whereas ischemic MMD patients did not.

**Conclusion:**

Patients with low levels of pre-anastomosis baseline CBF induce a dramatic increase in post-anastomosis and show a high risk of postoperative CHS.

## 1 Introduction

Moyamoya disease (MMD) is a chronic cerebrovascular disorder characterized by progressive stenosis or occlusion of the intracranial vessels.^1^^,^^2^ Revascularization surgery, specifically the superficial temporal artery (STA)—middle cerebral artery (MCA) bypass, is an effective method to improve blood flow perfusion.^3^^,^^4^ Despite the long-term efficacy of surgical revascularization,^5^ there is a concern regarding transient neurological deterioration caused by cerebral hyperperfusion or delayed intracranial hemorrhage after the surgery.^6^ Techniques such as fluorescence imaging of indocyanine green (ICG) offer real-time insights, enabling surgeons to navigate the complex cerebral vasculature with increased precision and adapt surgical strategies dynamically.^7^^,^^8^ Pioneering studies, such as those by Zhang et al.,^9^ demonstrated that the change in cerebral blood volume (CBV) and cerebral blood flow (CBF) correlated with the occurrence of cerebral hyperperfusion syndrome (CHS) using ICG video angiography. Similarly, Yang et al.^10^ found that the change in microvascular transit time greater than 2.6 s was an independent predictor of CHS. The recent application of intraoperative laser speckle contrast imaging (LSCI) by Tao et al.^11^ monitored blood flow changes using LSCI and reported that the cortical microvascular density >0.217 and the change of cerebral blood flow >1.985 were the risk factors for cerebral hyperperfusion.

Despite these advances, a knowledge gap persists regarding the underlying physiological or pathological factors driving the changes in cerebral blood flow post-bypass surgery. Baseline pre-anastomosis CBF can be influenced by various factors, including hemorrhagic and ischemic MMD subtypes and the patient’s sex,^12^^–^^14^ complicating preoperative assessment and prognostication. The relationship between pre-anastomosis and post-anastomosis CBF, particularly the impact of baseline CBF levels on postoperative CBF improvement is not yet fully understood. This suggests the need for further research into the risk factors of CHS.

We measured the cortical regional cerebral blood flow (rCBF) and regional blood flow structuring (rBFS) during side-to-side (S-S) STA-MCA bypass surgery in a cohort of adult MMD patients (n=48) using LSCI. The influences of MMD subtypes (hemorrhagic or ischemic) and patient sex on the microvascular response of STA-MCA bypass surgery were investigated. Statistical analysis suggested significant baseline rCBF differences between patients who developed CHS postoperatively and those who did not.

## 2 Materials and Methods

### 2.1 Patients with MMD

The surgeries included in this study were conducted at the Department of Neurosurgery, Zhongnan Hospital of Wuhan University. The study retrospectively analyzed MMD patients who underwent side-to-side superficial temporal artery-middle cerebral artery (SS-STA-MCA) bypass surgery performed by the same neurosurgeon (J.Z.) between October 2021 and October 2022. Diagnosis of MMD in all the patients was established with digital subtraction angiography (DSA).^8^ All patients met the diagnostic criteria set by the Research Committee on Spontaneous Occlusion of the Circle of Willis, Ministry of Health, Labour and Welfare, Japan.

The study protocol received approval from the institutional review board at Zhongnan Hospital of Wuhan University and was conducted by the revised 1983 Declaration of Helsinki. Written informed consent was obtained from all patients.

Inclusion criteria were as follows: (1) diagnosis of MMD according to the MMD diagnostic guidelines, (2) completion of LSCI, (3) no history of vascular intervention, and (4) adult MMD patients. Forty-eight patients met the eligibility criteria, as indicated in Table 1.

**Table 1:** Information on clinical data of adult patients with MMD.

	Ischemic (n=29)	Hemorrhagic (n=19)	P value
Age (year)	50.66±10.27	50.68±9.17	0.76
Sex	—	—	0.32
Male	15	12	—
Female	14	7	—
Suzuki stage	—	—	0.54
II	4	4	—
III	10	6	—
IV	10	8	—
V	5	1	—

### 2.2 Surgical Procedures

The surgical technique employed for S-S STA-MCA anastomoses followed the standard procedure outlined in previous articles.^8^ The patient is positioned supine, with the head rotated toward the contralateral side. Following a frontotemporal craniotomy, the STA-MCA (M4) anastomosis is performed. Intraoperative confirmation of patency is achieved using indocyanine green video angiography (ICG-VA), and LSCI recordings are captured before and after the anastomosis. Throughout the surgery, mean arterial pressure is maintained at a constant level of 90 to 100 mmHg, and the end-expiratory carbon dioxide (${\mathrm{CO}}_{2}$) concentration is maintained between 38 and 42 mmHg. Patients were identified as experiencing symptomatic hyperperfusion when they presented with transient neurological deficits (TNDs) manifesting as a constellation of symptoms, including but not limited to aphasia, limb numbness, diminished muscle strength, hemiparesis, seizures, dysarthria, intense headaches, and facial paralysis.^15^^,^^16^ These manifestations, indicative of the regions impacted by postoperative hyperperfusion, were meticulously evaluated and recorded by an experienced neurosurgeon.

### 2.3 Instrumentation of LSCI

In our study, LSCI was performed using a laser speckle device (SIM BFI HR Pro, SIM Opto-Technology Co., Ltd., Wuhan, China).^17^^–^^19^ The instrument operates at a laser wavelength of 785 nm, with an operating distance of 20 cm and a field of view of 42×31  mm. The camera exposure time was set at 20 ms. The instrument is positioned in advance before the procedure begins and does not interfere with the sterile hood of the microscope or the surgeon’s normal operation.

**Fig. 1:**
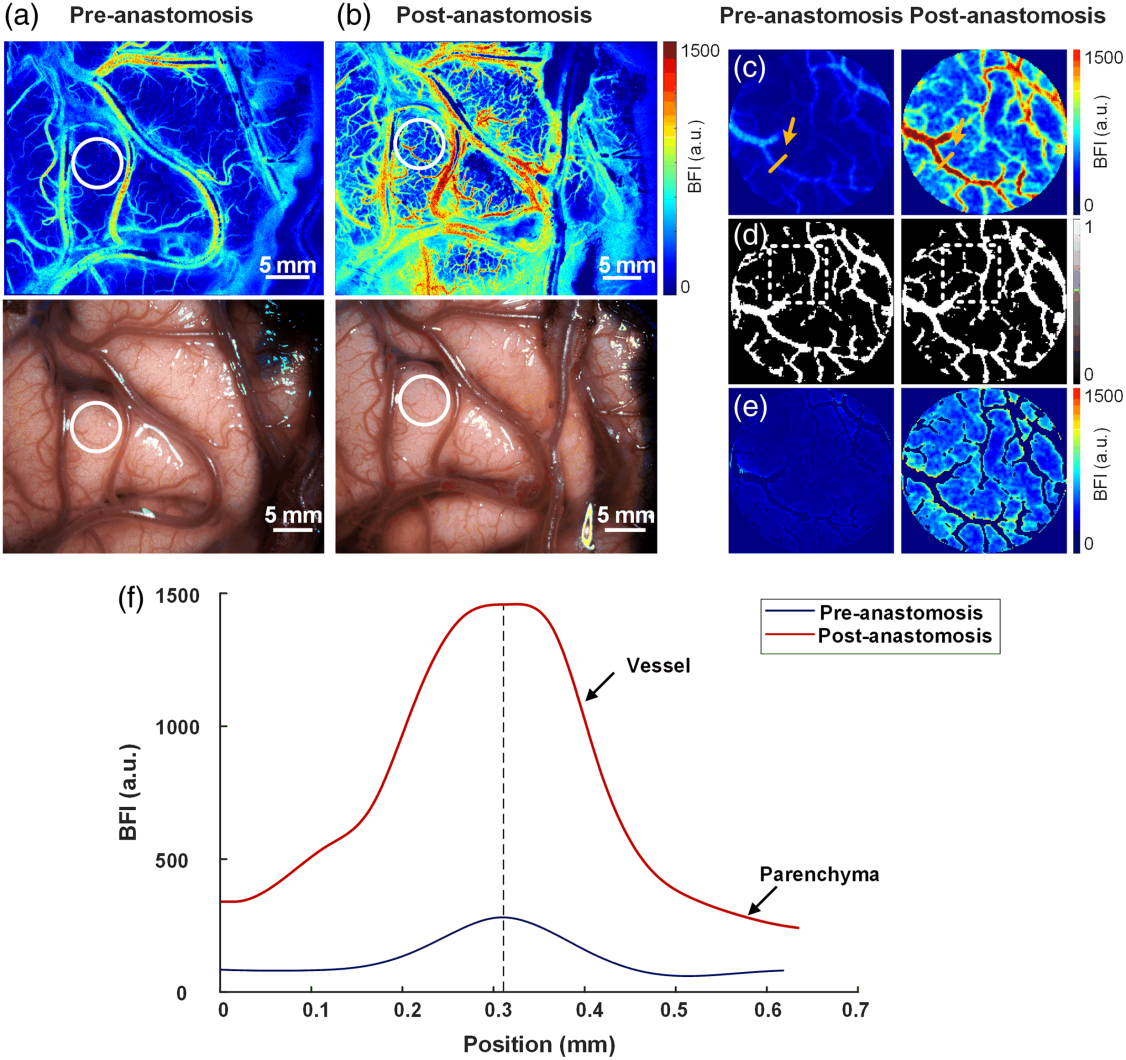
Illustration of intraoperative blood flow imaging and ROI analysis conducted using LSCI. (a) White light and BFI maps obtained within the operative field before anastomosis. (b) White light and BFI maps obtained within the operative field after anastomosis. (c) Magnified images of the ROI location (white circle) in panel (a). (d) Segmented vascular structures in panel (c). (e) Parenchyma BFI in panel (d). (f) Distribution of blood flow velocity index along the radial direction of the vessel in the yellow area of panel (c) before and after anastomosis.

During a craniotomy, the microscope is positioned above the patient at the neurosurgeon’s discretion, and the laser illumination is activated to display and record laser speckle blood flow images. The system can provide a real-time display of white light images [Figs. 1(a) and 1(b) upper side] and laser speckle blood flow images [Figs. 1(a) and 1(b) lower side] of the surgical area.

### 2.4 Image Analysis

Raw laser speckle images were processed by calculating the speckle contrast (K) within a 7×7  pixels window using the equation K=σ/⟨I⟩, where σ is the standard deviation of speckle intensity and ⟨I⟩ is the mean speckle intensity. The blood flow index (BFI), defined as calculated $1/K^{2}$, is commonly utilized as an approximation for blood flow perfusion.^20^^,^^21^

To quantify the changes in cortical microcirculation perfusion before and after the SS-STA-MCA anastomosis, regions of interest (ROIs) of the same size were selected after data collection for all cases was completed, as depicted by the white circle in Fig. 1(a). To ensure consistency of the locations of the ROI across pre-anastomosis and post-anastomosis blood flow images, image registration was performed by carefully comparing the morphology of the blood vessels within the ROIs. Figure 1(c) corresponds to the ROI of Figs. 1(a) and 1(b). Figure 1(f) illustrates the radial direction of BFI along the section for the selected vessels before and after anastomosis indicated by the yellow arrow in Fig. 1(c). It is clearly shown that BFI exhibits a significant increase after anastomosis, encompassing both intravascular and parenchymal regions (maximum BFI: pre-anastomosis 272.72 versus post-anastomosis 1472.7). Furthermore, the vessel diameter in the same section also increased after anastomosis [Fig. 1(f)]. Using a single threshold to binarize the BFI led to an underestimation of the vascular structure when the CBF index was relatively low. Therefore, we utilized the Hessian Frangi algorithm to extract morphological changes and enhance the vascular structure of the vessels.^22^^,^^23^ The enhanced image was segmented using the same threshold value, allowing differentiation of the blood flow structure from the parenchymal region.

Figure 1(d) depicts the blood flow structure before and after anastomosis. Notably, new flow structures emerge after anastomosis [white dashed box in Fig. 1(d)]. The ratio of the blood flow structure area within the ROI to the total ROI area was designated as regional blood flow structuring (rBFS, $\mathrm{rBFS}=S_{\text{vessel}}/S_{\mathrm{ROI}}$); here, $S_{\text{vessel}}$ represents the area of the blood flow structure within the ROI, and $S_{\mathrm{ROI}}$ denotes the total area of the ROI. The parameter $\Delta {\mathrm{rBFS}}_{\text{Post}/\mathrm{Pre}}={\mathrm{rBFS}}_{\text{Post}}/{\mathrm{rBFS}}_{\mathrm{Pre}}$ serves as an indicator of vessel dilation following anastomosis.

Figure 1(e) displays the BFI of the cortical regions after excluding vascular structures. The average BFI in the parenchyma regions was defined as regional cerebral blood flow (rCBF, $\mathrm{rCBF}=\underset{\text{Parenchyma}}{\sum }\mathrm{BFI}/S_{\text{Parenchyma}}$); here, $S_{\text{Parenchyma}}$ represents the parenchyma area excluding the blood flow structure. The parameter $\Delta {\mathrm{rCBF}}_{\text{Post}/\mathrm{Pre}}={\mathrm{rCBF}}_{\text{Post}}/{\mathrm{rCBF}}_{\mathrm{Pre}}$ was introduced to characterize the ratio of changes in cortical perfusion.

### 2.5 Statistical Analysis

This study utilized IBM SPSS Statistics V.22.0 for statistical analysis. Continuous variables with normal distribution were presented as mean ± standard deviation; non-normal variables were reported as median (interquartile range). To compare the parameters before and after anastomosis within each patient, a paired t-test was utilized. Categorical variables were analyzed in contingency tables with Fisher’s exact test. To control the false discovery rate, the false discovery rate (FDR) correction was applied. Receiver operating characteristic (ROC) analysis identified optimal cutoffs of rCBF and $\Delta {\mathrm{rCBF}}_{\text{Post}/\mathrm{Pre}}$ for predicting CHS. Independent t-tests were performed for rCBF and rBFS across different subtypes and sexes, given that the data followed a normal distribution. Levene’s test was used to assess the equality of variances, and Welch’s correction was applied when variances were unequal. For non-normally distributed $\Delta {\mathrm{rCBF}}_{\text{Post}/\mathrm{Pre}}$ and $\Delta {\mathrm{rBFS}}_{\text{Post}/\mathrm{Pre}}$, the Mann-Whitney U-test was employed. Pearson correlation coefficients determined the strength of correlations between variables. Analysis of covariance assessed differences in slopes and intercepts of fitted curves. Results with p<0.05 were considered statistically significant.

## 3 Result

### 3.1 Perfusion Changes in the Cortical Microcirculation Before and After Anastomosis

**Fig. 2:**
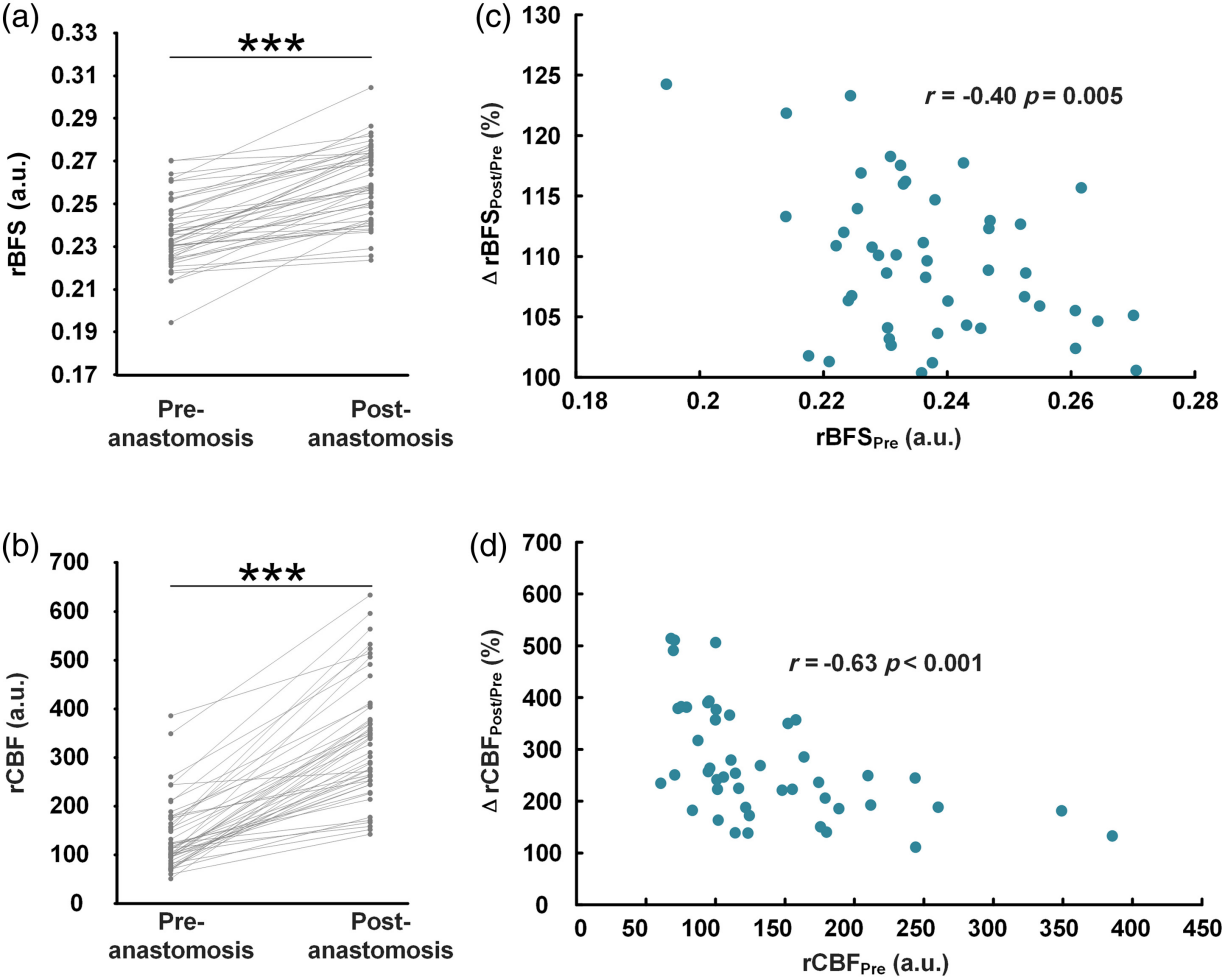
Changes in rBFS and rCBF before and after anastomosis in 48 patients. (a) There was a significant increase in rBFS after anastomosis compared to the pre-anastomosis state. (b) There was a significant increase in rCBF after anastomosis compared to before anastomosis. (c) ${\mathrm{rBFS}}_{\mathrm{Pre}}$ showed a significant negative correlation with $\Delta {\mathrm{rBFS}}_{\text{Post}/\mathrm{Pre}}$. (d) ${\mathrm{rCBF}}_{\mathrm{Pre}}$ showed a significant negative correlation with $\Delta {\mathrm{rCBF}}_{\text{Post}/\mathrm{Pre}}$. (*p<0.05, **p<0.01, ***p<0.001).

Statistical analysis was conducted on 48 patients to examine the changes in rBFS and rCBF before and after anastomosis. As shown in Fig. 2(a), a significant increase in rBFS was observed after anastomosis compared to before anastomosis (p<0.001). Figure 2(b) demonstrated a significant increase in rCBF (p<0.001). Both rBFS and rCBF exhibited increases after anastomosis in all patients. Figure 2(c) revealed a significant negative correlation between rBFSPre and $\Delta {\mathrm{rBFS}}_{\text{Post}/\mathrm{Pre}}$ (Pearson r=−0.40, p=0.005). Figure 2(d) showed a significant negative correlation between ${\mathrm{rCBF}}_{\mathrm{Pre}}$ and $\Delta {\mathrm{rCBF}}_{\text{Post}/\mathrm{Pre}}$ (Pearson r=−0.63, p<0.001).

Patients were grouped based on the presence or absence of CHS following surgery. Among these, seven patients exhibited transient symptoms of CHS postoperatively. According to the data presented in Table 2, a significant difference was observed in the ${\mathrm{rCBF}}_{\mathrm{Pre}}$ between patients with CHS and those who did not. $\Delta {\mathrm{rCBF}}_{\text{Post}/\mathrm{Pre}}$ also showed significant variation. Conversely, ${\mathrm{rCBF}}_{\text{Post}}$ and rBFS values showed no significant divergence between the two groups. Clinical variables such as sex, presence of hypertension, and the Suzuki stage did not reveal any significant differences between the groups.

**Table 2:** Comparison of basic characteristics and hemodynamic data.

	Univariate analysis			
	CHS (n=7)	Non-CHS (n=41)	P value	q value
Age	54.40±10.33	49.25±1.59	0.081	0.29
Female sex	4 (57.14)	17 (41.46)	0.87	0.87
Hypertension	2 (28.57)	16 (39.02)	0.74	0.87
Hemorrhagic onset	3 (42.85)	16 (39.02)	0.24	0.66
Suzuki stage	—	—	0.80	0.87
II	2 (28.57)	6 (14.63)	—	—
III	2 (28.57)	14 (34.14)	—	—
IV	1 (14.28)	17 (41.46)	—	—
V	2 (28.57)	4 (9.76)	—	—
${\mathrm{rCBF}}_{\mathrm{Pre}}$	97.10±34.68	147.47±76.05	0.0049	0.027
${\mathrm{rBFS}}_{\mathrm{Pre}}$	0.24±0.013	0.23±0.017	0.71	0.87
${\mathrm{rCBF}}_{\text{Post}}$	319.65±59.75	339.92±136.98	0.65	0.87
${\mathrm{rBFS}}_{\text{Post}}$	0.25±0.017	0.26±0.018	0.79	0.87
$\Delta {\mathrm{rCBF}}_{\text{Post}/\mathrm{Pre}}$	373.70 (222.50 496.75)	253.77 (183.00 334.00)	0.0048	0.027
$\Delta {\mathrm{rBFS}}_{\text{Post}/\mathrm{Pre}}$	109.24 (104.00 113.75)	109.62 (104.00 113.50)	0.71	0.87

Categorical variables, including sex, hypertension, subtype, and Suzuki stage, are presented as numbers (%).

Continuous variables with normal distribution were presented as mean ± standard deviation (SD); non-normal variables were reported as median (interquartile range).

The q-values indicate the adjusted P-values after using the FDR correction.

q<0.050 was considered significant.

**Fig. 3:**
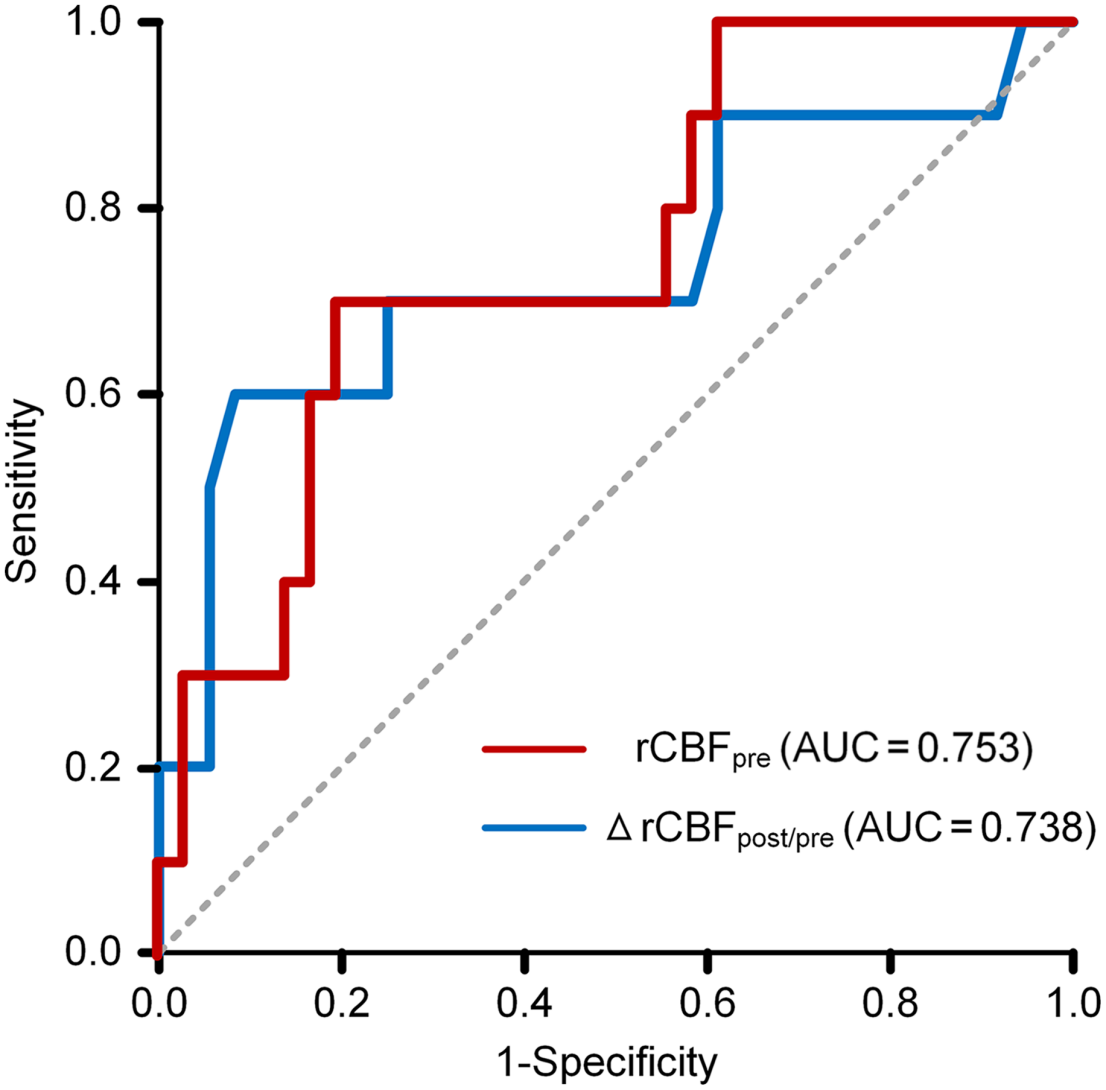
ROC curve of ${\mathrm{rCBF}}_{\mathrm{Pre}}$ and $\Delta {\mathrm{rCBF}}_{\text{Post}/\mathrm{Pre}}$ for predicting CHS.

Figure 3 illustrates the ROC curve analysis employed to evaluate the predictive power of ${\mathrm{rCBF}}_{\mathrm{Pre}}$ and $\Delta {\mathrm{rCBF}}_{\text{Post}/\mathrm{Pre}}$ concerning the onset of postoperative CHS. The optimal balance between sensitivity and specificity, as quantified by the Youden index, is achieved at cutoff values of 95.63 for ${\mathrm{rCBF}}_{\mathrm{Pre}}$ and 3.805 for $\Delta {\mathrm{rCBF}}_{\text{Post}/\mathrm{Pre}}$. The areas under the curve (AUC) for ${\mathrm{rCBF}}_{\mathrm{Pre}}$ and $\Delta {\mathrm{rCBF}}_{\text{Post}/\mathrm{Pre}}$ stand at 0.753 and 0.738, respectively.

### 3.2 Differences in Cortical Microcirculatory Perfusion Between Hemorrhagic and Ischemic

**Fig. 4:**
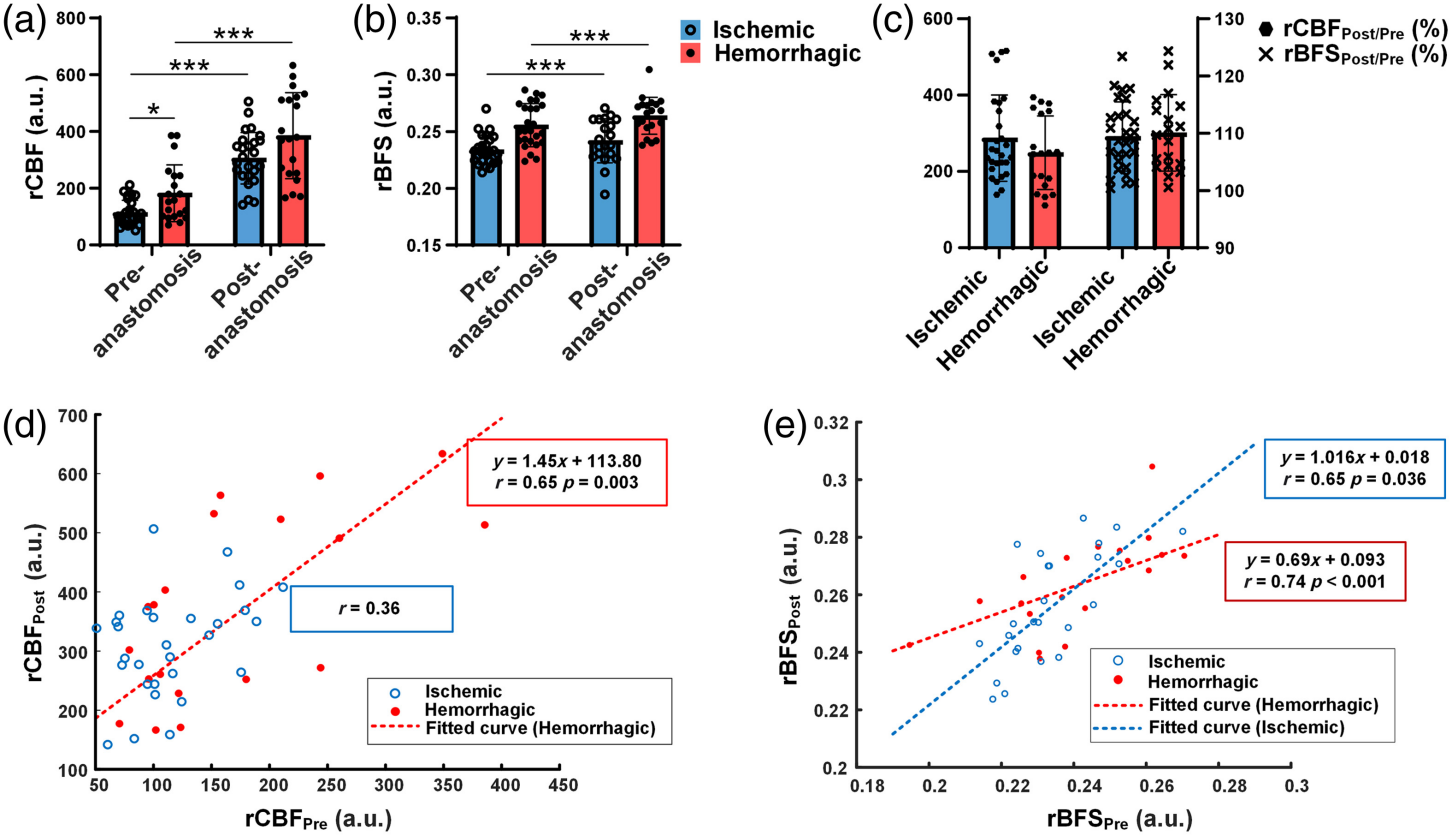
Comparison of rCBF and rBFS before and after anastomosis in hemorrhagic and ischemic MMD patients. (a) Statistics of rCBF for hemorrhagic and ischemic MMD patients. (b) Statistics of rBFS for hemorrhagic and ischemic MMD patients. (c) Statistics of $\Delta {\mathrm{rCBF}}_{\text{Post}/\mathrm{Pre}}$ and $\Delta {\mathrm{rBFS}}_{\text{Post}/\mathrm{Pre}}$ for hemorrhagic and ischemic MMD patients. (d) Curve fitting of the scatter plot illustrating the relationship between ${\mathrm{rCBF}}_{\mathrm{Pre}}$ and ${\mathrm{rCBF}}_{\text{Post}}$. (e) Curve fitting of the scatter plot illustrating the relationship between ${\mathrm{rCBF}}_{\mathrm{Pre}}$ and ${\mathrm{rCBF}}_{\text{Post}}$. (*p<0.05, **p<0.01, ***p<0.001).

Statistical analysis examined the changes in rCBF and rBFS before and after anastomosis in patients with hemorrhagic and ischemic MMD in SS-STA-MAC. Figure 4 illustrates that rCBF and rBFS increased significantly after anastomosis in both ischemic and hemorrhagic MMD patients (ischemic rCBF, p<0.001; ischemic rBFS, p<0.001; hemorrhagic rCBF, p<0.001; hemorrhagic rBFS, p<0.001).

In Fig. 4(a), the ischemic ${\mathrm{rCBF}}_{\mathrm{Pre}}$ was significantly lower than the hemorrhagic ${\mathrm{rCBF}}_{\mathrm{Pre}}$ (ischemic 116.26±41.57 versus hemorrhagic 122.35±68.58, p=0.036). However, there was no significant difference in rCBF between hemorrhagic and ischemic patients post-anastomosis. In Figs. 4(b)–4(c), there were no significant differences in rBFS between ischemic and hemorrhagic MMD patients.

Curve fitting was performed to analyze the relationship between ${\mathrm{rCBF}}_{\mathrm{Pre}}$ versus ${\mathrm{rCBF}}_{\mathrm{Post}}$ and ${\mathrm{rBFS}}_{\text{Pre}}$ versus ${\mathrm{rBFS}}_{\text{Post}}$ in patients with ischemic and hemorrhagic MMD [Figs. 4(d)–4(e)]. The Pearson correlation coefficient (r) was calculated and tested for significance. In Fig. 4(d), it can be observed that there was no significant correlation between ${\mathrm{rCBF}}_{\mathrm{Pre}}$ and ${\mathrm{rCBF}}_{\mathrm{Post}}$ in ischemic patients (r=0.36), whereas a significant correlation was found in patients with hemorrhagic MMD (r=0.65, p=0.003). The slopes of the two curves differed significantly (p<0.001), and the distribution of rCBF was more concentrated in patients with ischemic MMD compared to those with hemorrhagic MMD.

In Fig. 4(e), a significant correlation was observed between ${\mathrm{rBFS}}_{\mathrm{Pre}}$ and ${\mathrm{rBFS}}_{\text{Post}}$ in both hemorrhagic and ischemic MMD patients (ischemic, r=0.65, p<0.001; hemorrhagic, r=0.74, p<0.001). The slope of ${\mathrm{rBFS}}_{\text{Post}}$ in ischemic patients was significantly higher than that in hemorrhagic patients (ischemic, 1.016; hemorrhagic, 0.70; p<0.001). A higher rBFS slope indicates greater changes in ${\mathrm{rBFS}}_{\text{Post}}$.

### 3.3 Differences in Cortical Microcirculatory Perfusion Between Males and Females

**Fig. 5:**
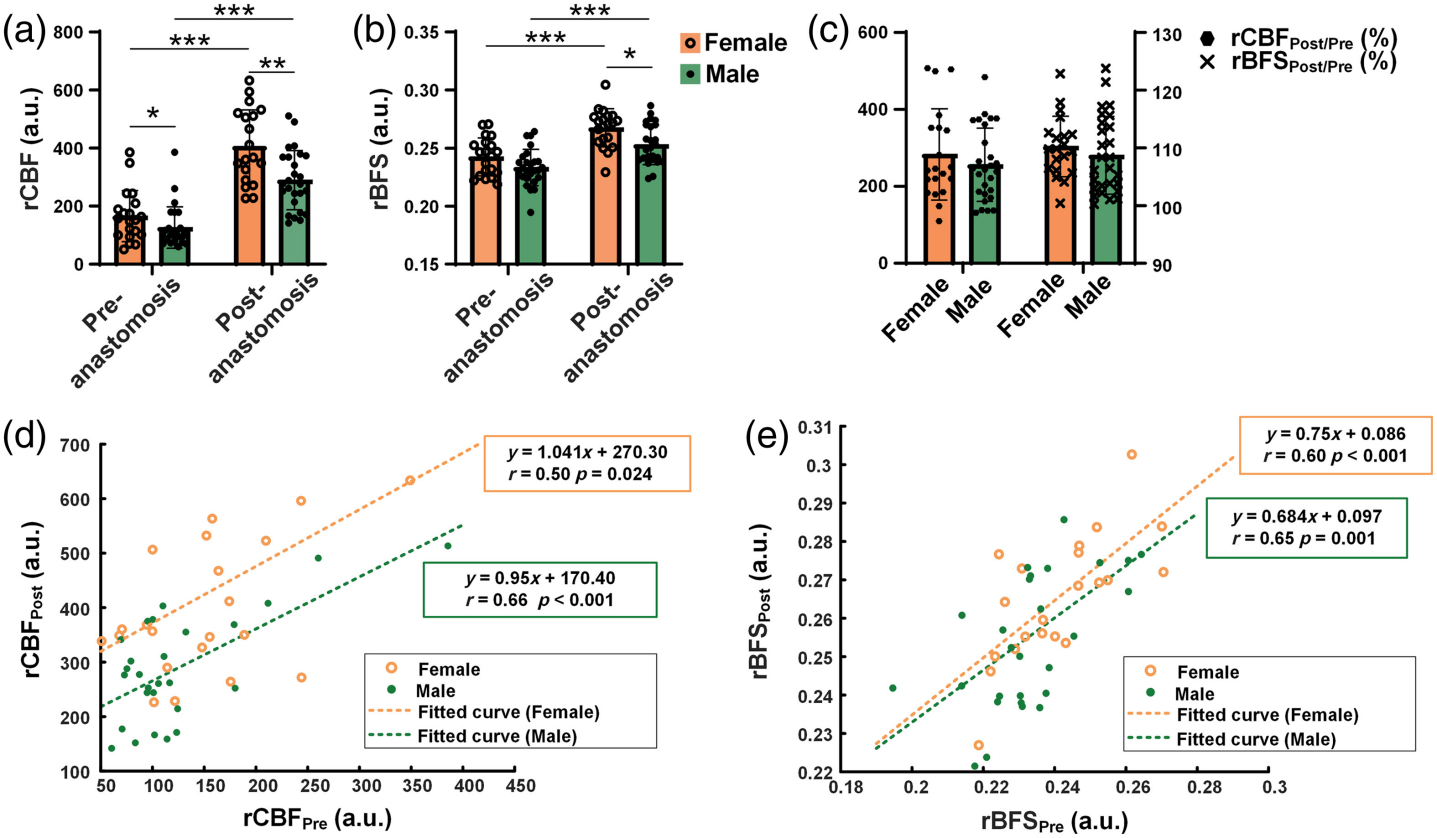
Comparison of rCBF and rBFS before and after anastomosis in male and female MMD patients. (a) Statistics of rCBF before and after anastomosis for male and female MMD patients. (b) Statistics of rBFS before and after anastomosis for male and female MMD patients. (c) Statistics of $\Delta {\mathrm{rCBF}}_{\text{Post}/\mathrm{Pre}}$ and $\Delta {\mathrm{rBFS}}_{\text{Post}/\mathrm{Pre}}$ for male and female MMD patients. (d) Curve fitting of the scatter plot illustrating the relationship between ${\mathrm{rCBF}}_{\mathrm{Pre}}$ and ${\mathrm{rCBF}}_{\text{Post}}$. (e) Curve fitting of the scatter plot illustrating the relationship between ${\mathrm{rCBF}}_{\mathrm{Pre}}$ and ${\mathrm{rCBF}}_{\text{Post}}$. (*p<0.05, **p<0.01, ***p<0.001).

Figure 5 presents the statistical analysis of rCBF and rBFS before and after the anastomosis in adults with MMD of different sexes. In Fig. 5(a), both sexes showed a significant increase in rCBF and rBFS after surgery (p<0.001 for both sexes). Female patients had significantly higher rCBF than male patients (p=0.043). After surgery, female patients continued to have significantly higher rCBF than males (p=0.035). In Fig. 5(b), there was no significant difference in ${\mathrm{rBFS}}_{\mathrm{Pre}}$ between male and female patients (0.24±0.015 in females versus 0.24±0.019 in males). However, after the anastomosis, rBFS was significantly higher in females than in males (0.27±0.014 in females versus 0.26±0.021 in males; p=0.005). In Fig. 5(c), there were no significant differences in $\Delta {\mathrm{rBFS}}_{\text{Post}/\mathrm{Pre}}$ and $\Delta {\mathrm{rCBF}}_{\text{Post}/\mathrm{Pre}}$ between sexes ($\Delta {\mathrm{rBFS}}_{\text{Post}/\mathrm{Pre}}$: 110.22±5.38 in females versus 108.99±6.84 in males; $\Delta {\mathrm{rCBF}}_{\text{Post}/\mathrm{Pre}}$: 305.08±143.34 in females versus 259.78±96.42 in males).

Curve fitting was performed for ${\mathrm{rCBF}}_{\mathrm{Pre}}$ versus ${\mathrm{rCBF}}_{\text{Post}}$ and ${\mathrm{rBFS}}_{\mathrm{Pre}}$ versus ${\mathrm{rBFS}}_{\text{Post}}$ for both sexes [Figs. 5(d)–5(e)]. The Pearson correlation coefficient (r) was calculated and tested for significance, and all four curves showed significant correlations. In Figs. 5(d)–5(e), the fitted curves of rCBF and rBFS for females had higher constant terms than those for males.

## 4 Discussion

ICG fluorescence imaging has emerged as a prominent method for evaluating cortical perfusion changes during bypass surgery, utilizing intraoperative hemodynamic parameters such as CBV, CBF, and microvascular transit time. However, as emphasized by Miller et al.,^24^ the temporal resolution of ICG imaging may prove inadequate for capturing rapid fluctuations in microcirculatory blood flow, whereas the presence of image artifacts, including motion blur, specular reflections, and background noise, can introduce inaccuracies in blood flow calculations. These challenges are particularly pronounced in the dynamic and demanding context of surgical environments. By contrast, LSCI has emerged as a promising approach. LSCI does not require the use of contrast agents and offers continuous blood flow monitoring capability, rendering it well-suited for real-time monitoring during surgical procedures, attracting increasing applications in neurosurgeries, including arteriovenous malformation embolization,^17^ cerebral aneurysm clipping,^25^ brain tumor resection,^26^^,^^27^ and STA-MCA bypass in MMD patients.^11^^,^^28^ Pioneering studies by Tao et al.^11^ and Hecht et al.^28^ have underscored the utility of LSCI in intraoperative monitoring and hemodynamic assessment during STA-MCA bypass surgery for MMD.

This study investigates how pre-anastomosis baseline CBF levels correlate with the changes in rCBF after STA-MCA anastomosis, and the onset of CHS. CHS is mostly caused by impaired cerebrovascular autoregulation of intracranial arteries, increased vascular permeability, and the sudden increase of blood input from the bypass artery.^29^ Previous reports indicate that various hemodynamic factors, such as hemodynamic sources of the PSCAs from the MCA,^16^ preoperatively increased oxygen extraction fraction,^30^ postoperative CBV increase,^9^^,^^31^ microvascular transit time,^10^ and the change of cerebral blood flow,^11^ are associated with CHS. While the exact causes of CHS remain controversial, excessively increased focal CBF is generally accepted as a leading factor.

A key finding of the present study is the negative correlation between the increase in $\Delta {\mathrm{rCBF}}_{\text{Post}/\mathrm{Pre}}$ and pre-anastomosis ${\mathrm{rCBF}}_{\mathrm{Pre}}$, mirrored in the $\Delta {\mathrm{rBFS}}_{\text{Post}/\mathrm{Pre}}$. The AUC for ${\mathrm{rCBF}}_{\mathrm{Pre}}$ and $\Delta {\mathrm{rCBF}}_{\text{Post}/\mathrm{Pre}}$ stood at 0.753 and 0.738, respectively. Patients with poorer cerebrovascular reactivity are known to have a higher risk for CHS.^32^ In this study, patients with lower baseline rCBF levels were more likely to develop CHS, potentially due to compromised cerebral autoregulation. In individuals with low baseline CBF, the sudden excessive increase in blood flow may overwhelm the cerebral vasculature’s ability to regulate and distribute blood flow, leading to hyperperfusion and subsequent neurological symptoms. This emphasizes the importance of using LSCI to closely monitor patients with lower baseline rCBF levels, allowing for earlier interventions that may reduce perioperative complications.

Some studies have indicated that patients with hemorrhagic MMD are susceptible to CHS,^32^ whereas others suggest that the onset of ischemia or hemorrhage has no significant effect on the incidence of postoperative CHS.^33^ Existing literature suggests that patients with ischemic MMD often have a longer natural history compared to those with hemorrhagic MMD.^34^^,^^35^ Long-term ischemia contributes to vascular vulnerability. Histopathological differences have also been observed between different types of MMD.^14^^,^^36^ While this study did not confirm the association between the MMD subtype and postoperative CHS, we identified a significant correlation between ${\mathrm{rCBF}}_{\mathrm{Pre}}$ in patients with hemorrhagic MMD, indicating consistent blood flow improvement after anastomosis. This correlation was absent in patients with ischemic MMD, suggesting a different response to treatment. In addition, the slope of ${\mathrm{rBFS}}_{\text{Post}}-{\mathrm{rBFS}}_{\mathrm{Pre}}$ fitted curve was lower in hemorrhagic than in ischemia MMD patients, indicative of smaller diastole in cortical microvessels. We postulate that patients with hemorrhagic MMD may exhibit a shorter natural history and better vascular elasticity, resulting in smaller changes in cerebral cortical microvessel diameter and more consistent alterations in perfusion, as observed in our study.

For MMD patients of different sexes, perfusion of the hippocampus and prefrontal cortex was better in females than in males.^14^ Our study is consistent and additionally complements that post-anastomosis rCBF and rBFS were significantly higher in females than in males. This suggests that the pre-anastomosis baseline rCBF level might have different implications for patients of different sexes undergoing STA-MCA bypass surgery. Further, the sex differences in hemodynamic parameters observed in our study are not influenced by the individuals who developed CHS. Factors such as hormonal influences,^37^ differences in cognitive function,^38^ and genetic predispositions^39^ might play a role. The underlying reasons for sex differences are not entirely clear and warrant further investigation.

We suggest that the pre-anastomotic CBF might be incorporated as a potential factor in strengthening perioperative management, and patients with low baseline rCBF level pre-anastomosis may need at least 24 h of sedation in addition to strict control of blood pressure and maintained body fluid balance. We expect to reduce perioperative complications through these managements and intend to assess whether treatment outcomes are improved by screening patients at increased risk for postoperative CHP using LSCI and enhancing perioperative management in the future. In addition, considering the complex hemodynamics in MMD patients and the limitations of LSCI imaging depth, integrating data from SPECT and MRI may enable a detailed assessment of whole-brain hemodynamics. We hope that a comprehensive study of risk factors for post-treatment complications will lead to more effective perioperative management.

## 5 Conclusions

STA-MCA bypass surgery significantly improves cortical microcirculatory perfusion, particularly in patients with low baseline perfusion levels. Patients with low pre-anastomosis baseline CBF experience a dramatic increase in post-anastomosis CBF, which may present a high risk of postoperative CHS, warranting careful postoperative management.

## Data Availability

Data underlying the results presented in this paper are available upon reasonable request.
